# Determination of Phenolic Content and Antioxidant Activity in Khabarovsk Soybean Cultivars

**DOI:** 10.3390/molecules31152671

**Published:** 2026-07-31

**Authors:** Andrei Kholodkov, Alexey Stepanov, Natalja Tsimbalist, Mayya Razgonova

**Affiliations:** 1 Far-Eastern Agriculture Research Institute, 680521 Vostochnoe, Russia; stepanfx@mail.ru; 2 Central Research Laboratory, Department of Biochemical Research Methods, Far Eastern State Medical University, 680000 Khabarovsk, Russia; kfnat@yandex.ru; 3 N.I. Vavilov All-Russian Institute of Plant Genetic Resources, 190031 Saint Petersburg, Russia; m.razgonova@vir.nw.ru; 4 Advanced Engineering School, Institute of Biotechnology, Bioengineering and Food Systems, Far Eastern Federal University, Fr. Russian, Ajax, 10, 690922 Vladivostok, Russia

**Keywords:** soybean, *Glycine max* (L.) Merr., above-ground parts, seeds, flavonoid content, metabolic profile, antioxidant activity, HPLC-MS/MS

## Abstract

In this study, total phenolic content (TPC), total flavonoid content (TFC), and antioxidant activity (I) were determined in eight soybean cultivars. Assays were conducted on above-ground parts (AGP) and seeds. Peak areas (S_TIC+_ and S_TIC−_) of major metabolites in the total ion current (TIC) chromatogram, obtained by HPLC–MS/MS, were also calculated. TPC in AGP and seeds ranged from 5.8% to 10.8% and from 2.2% to 4.4%, respectively. TFC was 0.3–1.2% and 0.02–0.07% for AGP and seeds, respectively. The coefficient of variation of I was 42% for AGP and 45% for seeds. The highest I in AGP was observed in Khabarovskij yubilyar (56.5%) and Kobra (54.3%), while that of seeds was observed in Kobra (47.5%). A positive correlation was found in AGP between I and TPC (0.75), as well as between I and S_TIC+_ (0.81). Strong significant correlations were found in seeds between I and TPC (0.90), TFC (0.83), S_TIC+_ (0.78), and S_TIC−_ (0.87). Leaf TPC, TFC, and I of the eight cultivars showed no correlation with AGP. These findings indicate that conducting targeted studies on AGP and seeds of food and vegetable varieties can help establish sustainable breeding programs for developing functional food products and animal feed.

## 1. Introduction

Soybeans (*Glycine max* L. Merr.) accounted for the largest share of the total harvested area of oil crops (41%) in 2024. Soybean production increased by 50% over 14 years, while that of rapeseed grew by 46%, despite a decline between 2023 and 2024. The Americas produced the majority of soybeans, with Brazil, the United States of America, and Argentina collectively accounting for 78% of global production [1].

Over the past decade, the area planted with soybeans in the Russian Federation has increased 2.5-fold, reaching 3.7 million hectares by 2023, while the gross harvest increased 4.2-fold to 6.7 million tons. Domestic soybean breeding and seed production are concentrated in the Central and Far Eastern regions. Between 2013 and 2023, state-funded breeding research institutions registered more than 120 soybean genotypes [2]. The primary objective of soybean breeding is to develop varieties that are resistant to environmental stressors, exhibit high productivity, and contain high levels of protein, fat, and essential micro- and macronutrients. In addition to these indicators, the nutritional value of soybean varieties is influenced by the presence of phenolic compounds with antioxidant activity in the plant.

Economic growth and technological progress have fueled demands for products that promote health and reduce the risk of disease. Most consumers prefer substances derived from natural sources over synthetic alternatives. In this context, soybean is recognized as a major source of flavonoids, encompassing a wide range of subclasses such as isoflavones, flavonols, flavanones, flavones, flavanols, and chalcones, as well as other minor groups, highlighting its metabolic diversity [3,4]. These characteristics highlight the importance of soybean as a functional food and nutraceutical crop. Flavonoids are a structurally diverse group of secondary metabolites that play a vital role in plant biology and human health. These polyphenolic compounds possess a unique C6-C3-C6 skeleton, which contributes to plant pigmentation, defense mechanisms, and protection against ultraviolet radiation. In humans, flavonoids function as potent antioxidants and exhibit anti-inflammatory, antimicrobial, and cardioprotective properties [5,6]. Flavones such as luteolin and apigenin have been shown to inhibit inflammatory pathways and reduce the risk of chronic diseases [7]. Daidzein, genistein, and glycitein are the main isoflavones in soybean, along with their glycoside forms and corresponding acetyl and malonyl derivatives. Among these 12 isoflavones, the glucoside and malonyl forms of daidzein and genistein are the most abundant in unprocessed soybean seeds [8].

Most studies examining the phenolic composition and antioxidant activity of soybeans focus on raw seeds. Researchers employ a wide range of physicochemical methods to assess the phenolic content and antioxidant activity in soybean seed extracts. This focus is particularly important given the crop’s widespread use as food and as animal feed. Cavaliere et al. identified 66 phenolic compounds using a liquid chromatography-electrospray ionization-tandem mass spectrometry (LC/ESI–MS/MS) system [9]. Iqbal et al. isolated 13 target flavonoid aglycones using a similar method and demonstrated the varietal and spatial variability of their content [10]. Cvejić et al. quantified the major soy isoflavones in 20 soybean varieties using HPLC [11]. Lee et al. examined the link between seed coat color and the concentration of specific isoflavone groups [12]. Malenčić et al. studied how seed coat color influences the total content of phenols, anthocyanins, flavonoids, and tannins, as well as antioxidant capacity [13]. Additional studies have evaluated flavonoid content in soybean seed coats and embryos [14]. Other researchers have proposed analytical methods and performed quantitative analyses of isoflavones and rutin in the seeds, roots, and leaves of Brazilian soybean varieties [15]. Antioxidant properties have also been assessed in seed extracts from cultivated and wild soybeans using DPPH and ABTS assays [16].

A substantial proportion of soybeans is allocated to the animal feed sector, human consumption, nutraceuticals, and other industrial applications. Soybean-derived meals are commonly classified as fermented or non-fermented. Traditional non-fermented soy-based foods include fresh soybeans, soy nuts, soybean sprouts, soymilk, whole dried soybeans, whole soybean meal, and soymilk-derived products such as yuba, tofu, and okara. Fermented soy-based foods include miso, soy sauces, tempeh, natto, and fermented soymilk products [17]. Soybean molasses is produced from seeds for use in animal feed applications. The main phytochemical components of soybean molasses are isoflavones, saponins, phenolic acids, phospholipids, phytosterols, phytates, ω-3 fatty acids, and leucoanthocyanins [18]. Soybean molasses can be sprayed onto soybean meal in a desolventizer–toaster or mixed with soybean hulls and incorporated into liquid feed rations [19]. Well-known soybean processors in Brazil produce approximately 220 tons of soybean molasses daily [20].

In addition to seeds, the composition of the above-ground parts (AGP) of soybeans, e.g., leaves, as well as by-products of seed processing, e.g., pod hulls, has been studied for potential applications in dietary supplements and food products. Previous studies have shown that soybean leaves contain a wide variety of phenolic substances that may help mitigate conditions such as diabetes and atherosclerosis [21,22]. Ho et al. analyzed soybean leaves and seeds for their flavonoid and isoflavone content [23]. Lin et al. examined the compositional profile, antioxidant activity, and anti-inflammatory properties of extracts from the leaves of a vegetable soybean variety [24]. Other studies have used HPLC–MS to characterize the flavonoid composition of soybean leaves and the ability of their extracts to inhibit pancreatic lipase activity [25].

Soybean hulls are also of interest, as they represent one of the main by-products of seed processing for animal feed. Several studies suggest the potential use of recalcitrant soybean hulls generated during processing as a source of high-value-added products for their dietary fiber, phenolics, and antioxidant capacity. Cabezudo et al. studied the phenolic profile of soybean hulls using HPLC–MS/MS, their antioxidant properties using the ABTS radical assay, and optimized extraction conditions for polyphenols [26]. Niño-Medina et al. compared the phenolic composition and biological activity of soybean and chickpea seed coats [27].

The phenolic composition and antioxidant capacity of soybean seeds, leaves, and seed pods have been extensively studied; however, research on the potential use of the AGP of the plant for functional foods, dietary supplements, and animal feed processing remains limited. However, when searching for the best variety for green forage, animal feed, straw, etc. processing, it is necessary to analyze the total AGP of different varieties. This approach is used in the pharmaceutical qualitative and quantitative analysis of plants, where the herb is considered the raw material. Many soybean genotypes have been registered in the Russian Far East, where three soybean breeding centers are located: the Amur Region, Primorsky Krai, and Khabarovsk Krai. To date, no systematic studies have been conducted across these centers to assess the varietal variability of Russian Far Eastern soybeans in terms of phenolic content or antioxidant activity. Such studies have been conducted in other regions of the world. For example, Josipović et al. examined 33 soybean varieties grown in Croatia [28]. Similar studies have also been reported by other researchers [29,30,31,32].

Research findings on soybean varieties developed in the Russian Far East may be of interest to manufacturers of soy-based products in both Russia and China. China imports large quantities of soybeans from Russia, and varieties developed in Khabarovsk Krai can be cultivated in China due to similar climatic conditions. Moreover, the cultivation of varieties with high antioxidant activity is promising for the production of food and feed additives. The objectives of the present study were to evaluate the phenolic content and antioxidant activity of and estimate the hypothetical differences between soybean varieties developed at the Khabarovsk Soybean Breeding Center. When conducting the targeted breeding of soybean varieties for use as animal feed (green forage, straw), it is reasonable to evaluate the antioxidant properties and bioactive metabolite content in whole AGP rather than analyze parts separately. It is rational to assess seeds for use as a functional food or soy-based food product. Eight soybean varieties from the Khabarovsk breeding program were examined. Total phenolic content, total flavonoid content, and antioxidant activity were determined using extracts of the AGP and seeds. The metabolic profile was also analyzed, and total ion current (TIC) chromatograms were constructed. Correlations between chromatographic characteristics and phenolic content, flavonoid content, and antioxidant activity (I) were determined.

## 2. Results

Proximate analyses of seeds of the eight soybean genotypes have been presented in Table 1.

It can be observed from Table 1 that the values of crude protein, nitrogen and moisture are not significantly different among eight soybean varieties (*p* > 0.05). In contrast, the fat content differed significantly (*p* < 0.05). The crude protein content ranged from 37.4% to 39.7% in Tasia and Khekhcirskaya, respectively. The difference between the highest and lowest fat content is 1.2-fold. The lowest nitrogen content was in Tasia and the highest was in Khekhcirskaya. Moisture content ranged from 6.32% to 6.65%. Table S1, Supplementary files, shows confidence probabilities for Tukey’s method (seeds; fat). Confidence probabilities for Tukey’s method revealed that there is mostly no significant difference in fat content between all varieties. Significant differences were revealed only in pairs Batya vs. Marinata (*p* < 0.05), Marinata vs. Zhemchuzhina vostoka (*p* < 0.01) and Zhemchuzhina vostoka vs. Tasia (*p* < 0.05).

Analysis of variance revealed significant differences in the content of biologically active compounds and antioxidant activity (I) in the AGP of soybean varieties (*p* < 0.05) (Table 2). The greatest varietal variability was observed in I (41.9%). The average total phenolic content (TPC) in the AGP was 9.2%; the lowest value was recorded in Marinata (5.8%), while the highest was recorded in Zhemchuzhina vostoka (10.8%). Total flavonoid content (TFC) in the AGP extracts ranged from 0.3% in Marinata to 1.2% in Zhemchuzhina vostoka, Kobra, and Khabarovskij yubilyar. I ranged from 16.1% in Marinata to 54.3% in Khabarovskij yubilyar. Table S2–S4, Supplementary files, shows confidence probabilities for Tukey’s method (AGP; TPC, TFC, I). Confidence probabilities for Tukey’s method revealed that the TPC in Marinata had a significant difference (*p* < 0.001) from other varieties. TFC content in Batya and Marinata also significantly differed from other varieties (*p* < 0.05 and < 0.001, respectively). The I values significantly differed from other varieties in Batya (*p* < 0.001) and Marinata (*p* < 0.001).

In seeds, a higher coefficient of variability was observed for TPC (23.9%), TFC (43.0%), and I (45.1%) than for AGP (Table 3). As in AGP, significant differences in metabolite content were detected (*p* < 0.05). The average TPC was three times lower (2.8%) than in AGP, with the highest value recorded in Kobra (4.4%) and the lowest in Soyuznaya 17 (2.2%). TFC ranged from 0.02% in Marinata to 0.07% in Kobra; the average value was 28-fold lower than in AGP (0.04%). The average I in seed extracts was also lower than that of AGP (25.5%). Among the varieties, the highest I was found in Kobra (47.5%), while the lowest was found in Batya (12.7%). Table S5–S7, Supplementary files, shows confidence probabilities for Tukey’s method (seeds; TPC, TFC, I). Confidence probabilities for Tukey’s method revealed significant differences from other varieties in TPC in Soyuznaya 17 (*p* < 0.001), Kobra (*p* < 0.001) and Khabarovskij yubilyar (*p* < 0.001). Kobra also had a significant difference in TFC (*p* < 0.001). Batya, Kobra and Khabarovskij yubilyar significantly differed in I (*p* < 0.001).

**Figure 1:**
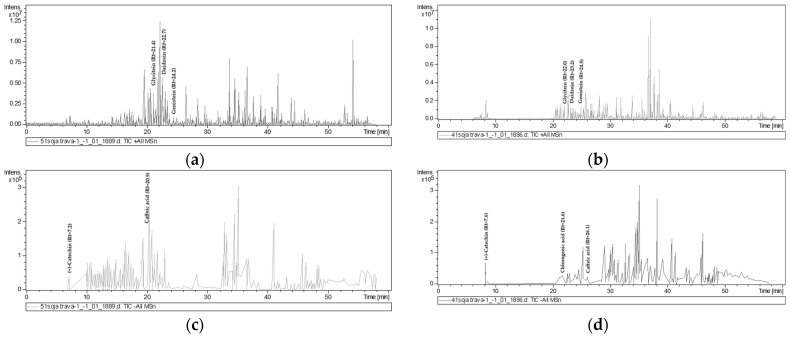
HPLC–ESI–MS/MS chromatograms of AGP extracts of Kobra and Zhemchuzhina vostoka varieties in negative and positive ionization modes: (**a**) Kobra, TIC+; (**b**) Zhemchuzhina vostoka, TIC+; (**c**) Kobra, TIC−; (**d**) Zhemchuzhina vostoka, TIC−.

HPLC–MS/MS was used to obtain chromatograms of soybean varieties. All identified compounds showed mass accuracy below 2 mDa. The total peak intensity of the metabolic profiles of AGP extracts was comparable to that of TIC chromatograms of seed extracts, while the total number of detected compounds in AGP chromatograms was higher (Figure 1). Overall, 22 significant positive ions (mAU × 10^7^ > 0.2) were identified in Kobra, 14 significant positive ions in Zhemchuzhina vostoka, 27 significant negative ions (mAU × 10^5^ > 0.5) in Kobra, and 16 significant negative ions in Zhemchuzhina vostoka.

**Figure 2:**
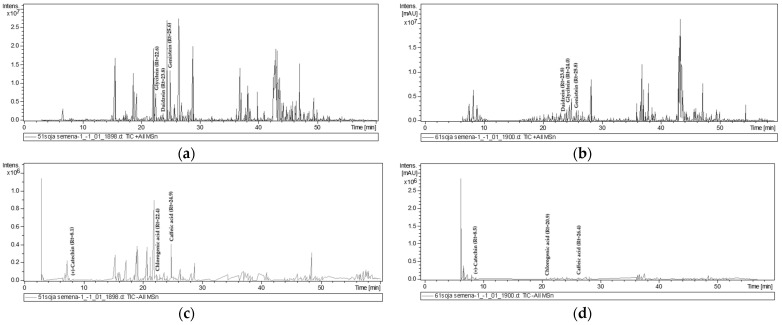
HPLC–ESI–MS/MS chromatograms of seed extracts of Kobra and Tasia varieties in negative and positive ionization modes: (**a**) Kobra, TIC+; (**b**) Tasia, TIC+; (**c**) Kobra, TIC−; (**d**) Tasia, TIC−.

Metabolic profiles of seed extracts were constructed for all varieties (Figure 2). The relative quantitative content of the identified metabolites, estimated based on the number, height, and area of the peaks, was higher in Kobra seeds than in Tasia seeds. Overall, 8 significant positive ions (mAU × 10^7^ > 0.5) were identified in Tasia, 18 significant positive ions in Kobra, 2 significant negative ions (mAU × 10^6^ > 0.2) in Tasia, and 6 significant negative ions in Kobra.

The chemical constituents were identified by comparing their retention index, mass spectra, and MS fragmentation with data from the literature and spectral libraries (confidence level 2). The widely used nomenclature system for flavonoid aglycones developed by Mabry and Markham has been applied to define the various A and B ring fragments generated by electron ionization [33]. The characteristic ion signals that distinguish isoflavones from other flavonoids are generated by the consecutive loss of up to four molecules of CO; H_2_O loss is also observed [34,35]. The three aglycones of well-known soy isoflavones—genistein, daidzein and glycitein—were identified in the AGP and seeds of all soybean varieties in positive mode (Table 4 and Table 5). The main ion [M + H]^+^ at *m*/*z* 255, daidzein, was observed within a similar retention time window (RTW) in both AGP and seeds, specifically 21.9–24.5 and 21.2–23.8, respectively. Genistein (*m*/*z* 271) was detected at RTW 23.2–25.7 in AGP and 25.2–27.3 in seed extracts. Glycitein, the main ion at *m*/*z* 285, was registered at RTW 21.3–22.7 and 22.6–24.2 in AGP and seeds, respectively. Consistent with findings from previous studies, product ions formed from [M + H] were also detected [9]. Isoflavone glycitein with base peak [M + H] ^+^ at *m*/*z* 285 when loss of 15 Da occurred first (*m*/*z* 270), followed by the intense loss of one CO molecule (*m*/*z* 242); the additional loss of the second CO molecule and OH˙ gave product ion with *m*/*z* 197. For genistein with base peak [M + H]^+^ at *m*/*z* 271, the loss of 18 Da (H_2_O) gave characteristic product ion *m*/*z* 253, and the loss of 56 Da (2CO) produced ion at *m*/*z* 215. Isoflavone daidzein base peak [M + H]^+^ at *m*/*z* 255 loss of H_2_O occurred first (*m*/*z* 237); the loss of two CO molecules gave the base peak at *m*/*z* 199.

The results of fragmentation in negative mode showed that all AGP and seed samples from the eight cultivars contained common organic acids, consistent with previous findings reported by Iqbal et al. (Table 6 and Table 7). The main ion [M + H]^+^ at *m*/*z* 289, (+)-Catechin, was registered within the RTW 6.6–8.8 in AGP and 8.0–9.7 in seeds. The main ion at *m*/*z* 353, identified as chlorogenic acid, was registered within the RTW 19.5–21.9 in AGP and 20.9–26.9 in seeds. Caffeic acid was also identified at *m*/*z* 179, within the RTW 20.9–27.3 in AGP and 24.3–26.4 in seeds. The major fragment ions (*m*/*z* 135) were produced by the loss of CO_2_ (44 Da) from the precursor ion [M − H]^−^ of caffeic acid. The MS/MS on the precursor *m*/*z* 353 ions, identified as chlorogenic acid, gave dominant product ions *m*/*z* 191. The (+)-catechin main ion at *m*/*z* 289 produced the major fragment ion (*m*/*z* 245) with the loss of one molecule of CO_2_.

Peak areas of total ion chromatograms of seeds and AGP in positive and negative ionization modes have been presented in Table 8.

Significant correlations were found between TPC and TFC (r = 0.94), as well as between the peak areas of S_TIC+_ and S_TIC−_ (r = 0.89) in AGP (Table 9). However, the correlation between S_TIC−_ and the other parameters was not statistically significant (*p* < 0.05). Antioxidant activity correlated with TPC and S_TIC+_ peak areas (r = 0.75 and r = 0.81, respectively). The peak areas of the chromatograms showed a strong correlation (r = 0.89).

In seed extracts, significant strong correlations were observed between I and both TPC and TFC, with correlation coefficients of 0.90 and 0.83, respectively (Table 10). Significant correlations were also found between TPC, TFC, I, and S_TIC+_ and S_TIC−_ (*p* < 0.05). The correlation between TFC and S_TIC+_ was not statistically significant (*p* < 0.05).

## 3. Discussion

### 3.1. Variation Among the Cultivars

Several studies have examined the varietal variability in phenolic content and antioxidant activity among soybean varieties [32,37,38,39,40,41]. For example, Abd Elhamid et al. reported significant varietal differences in the seed phenol content. The minimum and maximum TPC (*CV* = 17%) differed by 1.6-fold, whereas TFC (*CV* = 31%) exhibited a 2-fold difference. Antioxidant activity, measured using the DPPH assay, showed a 1.7-fold difference between the maximum and minimum values, with a coefficient of variation of 18%. Zhang et al. analyzed 60 soybean varieties with black seed coats. They noted a 12-fold difference in TFC and a 13-fold difference in I between the maximum and minimum values. Antioxidant activity showed a positive correlation with TPC.

Significant varietal differences were also observed in this study. The minimum and maximum TPC values (*CV* = 24%) in seeds differed by 1.5-fold. Those of TFC (*CV* = 43%) and I (*CV* = 45%) differed by 3.5-fold and 4-fold, respectively. The high coefficient of variation can be explained by the inclusion of the Kobra cultivar, which has black-colored seeds and exhibits significantly higher TPC and I. Conversely, variability in protein, nitrogen, and fat content was significantly lower. Therefore, the goal of variety breeding is to obtain genotypes that combine high protein or fat content with low varietal variability, are rich in phenols and flavonoids, and exhibit enhanced biological activity.

**Figure 3:**
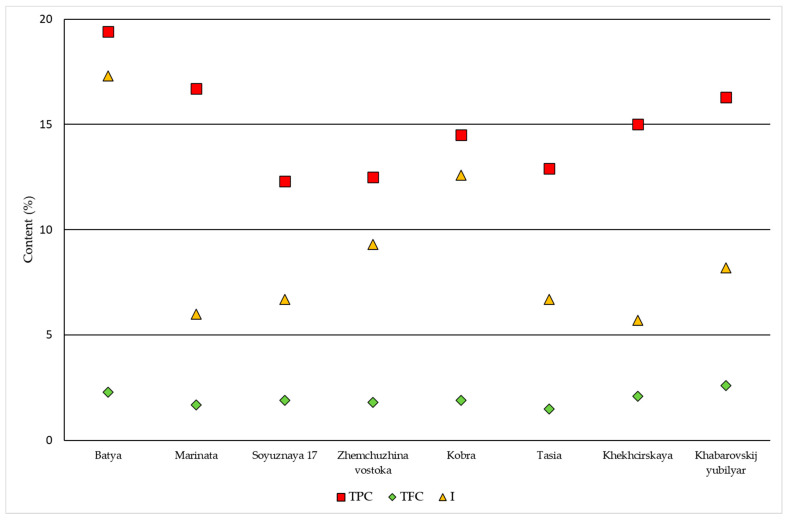
TPC, TFC and I of eight soybean cultivars leaves. All data are expressed as the mean (*n* = 3).

In the present study, the AGP of soybean plants exhibited significant varietal variability (*p* < 0.05). TPC ranged from 5.8% to 10.8%, representing nearly a 2-fold variation. The minimum and maximum TFC differed 4-fold, ranging from 0.3% to 1.2%. Antioxidant activity ranged from 16.1% to 56.5%, corresponding to a 3.5-fold difference. To test the hypothesis regarding the effect of foliage density on TPC, TFC, and I, we analyzed dried leaves from all varieties. The proportion of leaves (by weight) in all samples was 28% Marinata, 31% Kobra, 37% Khekhcirskaya, 39% Batya, 41% Soyuznaya 17, 43% Khabarovskij yubilyar, 48% Tasia, and 76% Zhemchuzhina vostoka. No significant correlations were found between this parameter and the metabolite content in dried leaves (Figure 3). The highest TPC was observed in Batya, while the lowest values were characteristic of Soyuznaya 17. The Tasia and Marinata cultivars exhibited the lowest TFC. Our findings confirm the assumption that assessing the content of biologically active substances in soybean above-ground plant material, including leaves, stems, and other tissues, is appropriate.

Significant differences were observed in the total metabolite content of AGP and seeds (*p* < 0.0001). Antioxidant activity also differed significantly (*p* < 0.05). Several previous studies have examined the phenolic profile and I of the leaves [42,43,44].

In the present study, Kobra is the only variety that produces black seeds; the others produce yellow seeds (Table 11). The phenolic content in Kobra was nearly twice that of the other varieties (4.44%). Similarly, TFC and I were also approximately twice as high as all other varieties except for Khabarovskij yubilyar (36.9%).

Previous studies analyzing the phenolic content and antioxidant activity of seeds with different seed coat colors have reported significant differences in total metabolite content and antioxidant activity among varieties [45,46]. Slavin et al. investigated the isoflavone content in varieties producing black, green, yellow, and brown seeds. The average total isoflavone content in black seeds (2.3 μmol/g) was significantly higher than in the other varieties (*p* < 0.05)—specifically, three times higher than in brown and yellow seeds (0.90 μmol/g) and twice as high as in green seeds (1.2 μmol/g). TPC was also significantly higher in black seeds and positively correlated with I (radical scavenging capacity) as measured by the ORAC and ABTS^•+^ methods. Xu et al. also studied yellow and black seeds and found that the TPC in black seeds was three times higher than in yellow seeds. Similarly, TFC was significantly higher in black seeds at five times the level observed in yellow seeds.

### 3.2. HPLC-MS/MS Analysis

#### 3.2.1. Determination of Individual Metabolites in Both Seeds and AGP

The characteristic isoflavone aglycones daidzein, genistein, and glycitein have been identified in Khabarovsk soybean varieties. Several studies have also reported the presence of these isoflavones in seeds [47,48]. Moreover, these compounds were detected in the AGP of all eight varieties, consistent with findings from other studies [44,49]. The data indicate the presence of additional metabolites; however, the number and intensity of peaks vary among varieties. This variation may be explained by differences in the relative proportions of aglycones and glycosides. Therefore, for the comparative assessment of Far Eastern varieties, we used the areas of significant peaks and their total values. AGP extracts contained more metabolite peaks than seeds. However, the total peak intensity in the metabolic profiles of AGP extracts was comparable to that observed in the TIC chromatograms of seed extracts.

#### 3.2.2. Comparative Assessment of Metabolites Content

Examples of using TIC chromatograms as reference chromatograms for the comparative assessment of metabolite content are presented in Chen et al., where chromatographic peaks from wild and cultivated soybean seed extracts were compared with control samples. Metabolic profiles were obtained for soybean varieties selected in Khabarovsk in both positive- and negative-ion modes. Data from AGP extracts showed a moderate correlation (r = 0.81) with antioxidant activity. In seeds, the total peak areas of chromatograms in positive- and negative-ion modes also showed moderate correlations (r = 0.73–0.87) with TPC, TFC, and I. No significant correlation was observed between TFC and S_TIC+_ in this study.

Abd-Alla et al. used relative peak areas from metabolite chromatograms to compare variability in the phytochemical composition of five soybean genotypes via GC/MS [50]. The total peak areas of the chromatograms for identified metabolites exhibited high variability (56%) and a strong positive correlation with I (r = 0.98) as measured using the DPPH assay. These findings are consistent with data from seeds of Khabarovsk soybean varieties, in which correlations between the total peak areas in both positive- and negative-ion modes and I were moderate (r = 0.78 and 0.87, respectively).

## 4. Materials and Methods

Eight Khabarovsk soybean cultivars (Khabarovskij yubilyar, Soyuznaya 17, Marinata, Tasia, Batya, Khekhcirskaya, Zhemchuzhina vostoka, and Kobra) were obtained from the Far Eastern Agriculture Research Institute, Khabarovsk Federal Research Center, Far Eastern Branch of the Russian Academy of Sciences, Khabarovsk, Russia. Photographs of seeds and AGP samples are shown in Figures S3 and S4, Supplementary Files. Soybean plants were grown in 2024 at the same experimental site in the Khabarovsk Krai (latitude 48°28′51.0″ N and longitude 135°15′02.0″ E). AGP samples of all cultivars were harvested in August 2024 during the R6 growth stage, and seed samples were harvested in October 2024 during the R8 growth stage. The origin and pedigree of all cultivars are provided in Table 11. Seed and AGP samples were collected from a single experimental site at one year, combined, dried, ground, and distributed to ensure that the samples were homogeneous.

### 4.1. Chemicals and Reagents

Analytical-grade reagents and ultrapure water were used for liquid chromatography (LC) and mass spectrometry (MS). Analytical grade aluminium chloride anhydrous, sodium carbonate anhydrous, 2,2-Diphenyl-1-picrylhydrazyl (DPPH) and Folin & Ciocalteu’s phenol reagent were purchased from CDH (New Delhi, India); HPLC-grade acetonitrile was purchased from Fisher Scientific (Southborough, UK); analytical grade anhydrous acetic acid, ethanol were purchased from Russia. Ultra-pure water was prepared by using a SIEMENS ULTRA clear (SIEMENS water technologies, Berlin, Germany).

### 4.2. Plant Material Preparation

Soybeans were manually dehulled to remove seed coats. Dehulled seeds (approximately 2 g) were milled and sieved to a particle size of 2 mm using a coffee grinder (Robert Bosch GmbH, Karlsruhe, Germany). The resulting soy meal was stored in the dark at 25 °C. Soybean AGP were also dried at 25 °C, ground using a coffee grinder to obtain flour with a particle size of less than 2 mm and stored under the same conditions.

### 4.3. Proximate Analysis

Soybean samples were analyzed in triplicate for crude protein, moisture, fat, and nitrogen content according to Association of Official Analytical Communities (A.O.A.C.) methods [51].

### 4.4. Phenolic Compounds Extraction

Extraction procedures were developed for quantitative analysis according to Benedec et al. with slight modifications [52]. A sample (2.0 g) of raw seeds or AGP was placed in a 250-mL flask and extracted using 100 mL of 70% ethanol (*v*/*v*) in a water bath equipped with a reflux condenser for 30 min. The extracts were filtered through a 12-µm paper filter and stored at 25 °C in the dark until further use.

### 4.5. Determination of Total Phenolic Content

TPC was determined via the Folin–Ciocalteu assay according to Spanos & Worlstad with slight modifications [53]. For the analysis, 1 mL of AGP extract or 2 mL of seed extract was mixed with 7.5 mL of distilled water, then 0.5 mL of Folin–Ciocalteu reagent. After 3 min, 1 mL of 20% sodium carbonate (Na_2_CO_3_) (*w*/*v*) solution was added. The contents were mixed and incubated in the dark at 25 °C for 1 h. The absorbance of the resulting green color was then measured at 720 nm using a UV-visible spectrophotometer (UV-2450, Shimadzu, Tokyo, Japan), with distilled water used as the reference solution. TPC was expressed as grams of gallic acid equivalents per 100 g of sample (g of GAE/100g of sample).

(1) $$\mathrm{TPC}\%=\frac{Abs720\times \;25\times \;100\times \;100}{a\times \;1\left(2\right)\;\times \;90\times \;(100-w)}$$

where *Abs*_720_ is absorbance in the presence of the test compound, *a* is the weight of soybean seeds or AGP sample, and *w* is the moisture content of soybean seeds or AGP.

### 4.6. Determination of Total Flavonoid Content

TFC was determined using a colorimetric method according Chandra et al. with slight modifications [54]. For the analysis, 2 mL of AGP extract or 10 mL of seed extract was placed in a 25-mL volumetric flask. Then, 3 mL of 2% ethanolic aluminum chloride (*w*/*v*) was added, and the mixture was diluted to volume using 70% ethanol (*v*/*v*). The mixture was then incubated in the dark at 25 °C for 30 min. A reference solution was prepared by placing 2 mL of AGP extract or 10 mL of seed extract in a 25-mL volumetric flask, adding 0.1 mL of anhydrous acetic acid, and diluting to volume using 70% ethanol (*v*/*v*). The optical density of the solution was measured at 410 nm using a UV-visible spectrophotometer (UV-2450, Shimadzu, Japan). TFC was expressed as grams of rutin equivalents per 100 g of sample (g of RE/100 g of sample).

(2) $$\mathrm{TFC}\%=\frac{Abs410\times \;25\times \;100\times \;100}{a\times \;2\left(10\right)\;\times \;260\times \;(100-w)}$$

where *Abs*_410_ is the absorbance of the test compound, *a* is the weight of the soybean seed or AGP sample, and *w* is the moisture content of soybean seeds or AGP.

### 4.7. Free-Radical Scavenging Activity (DPPH Assay)

The free-radical scavenging activity of soybean cultivars was determined using the DPPH assay according Xu et al. with slight modification [55]. DPPH was dissolved in 96% ethanol (*v*/*v*) to a concentration of 5.1 µM. A 3.9-mL aliquot of DPPH solution was mixed with 1 mL of seed extract in 70% ethanol or with 0.6 mL of AGP extract in 70% ethanol (*v*/*v*). The absorbance of the reaction mixtures was measured at 517 nm after 30 min of incubation at 25 °C in the dark to determine the amount of DPPH scavenged. The free-radical scavenging activity of the samples was expressed as the percentage inhibition of the DPPH free radical (I%), calculated as follows:

(3) $$I\%\;=\;\frac{Abs0-\;Abs1\;}{Abs0}\times 100$$

where *Abs*_0_ is the absorbance of the control and *Abs*_1_ is the absorbance in the presence of the test compound.

### 4.8. HPLC–MS/MS Analysis

HPLC–MS/MS analysis was conducted according Razgonova et al. [56]. A 2.0-g sample of raw seeds or AGP was placed in a 250-mL flask and extracted using 100 mL of 70% ethanol in a water bath equipped with a reflux condenser for 30 min. The extracts were filtered through a 12-µm paper filter and washed using 70% ethanol (*v*/*v*) into a round-bottomed flask. The filter was further washed with 10 mL of 70% ethanol, and the filtrates were combined. The solvent was evaporated, and the dry extract was dissolved in 3 mL of 70% ethanol (*v*/*v*). The solution was then diluted to volume using 70% ethyl alcohol (*v*/*v*) in a 5-mL volumetric flask and filtered through a Chromafil Xtra PA-45/25 syringe filter (Düren, Germany).

HPLC was performed using a Shimadzu LC-20 Prominence HPLC system (Shimadzu, Japan) equipped with a UV sensor and a Shodex (Tokyo, Japan) ODP-40 4E reverse-phase column for the separation of multicomponent mixtures. The gradient elution program was as follows: 0.01–4 min, 100% CH_3_CN; 4–35 min, 100–25% CH_3_CN; 35–50 min, 25–0% CH_3_CN; and 50–60 min, 0% CH_3_CN for column washing. The analysis was conducted using an ESI detector at wavelengths of 230 nm and 330 nm, and the column temperature was maintained at 17 °C. The injection volume was 1 µL.

MS analysis was performed using an amaZon ion trap SL spectrometer (Bruker Daltonics, Bremen, Germany) equipped with an ESI source operating in negative-ion mode. The optimized parameters were as follows: ionization source temperature: 70 °C, gas flow: 4 L/min, nebulizer gas (atomizer) pressure: 7.3 psi, capillary voltage: 4500 V, end plate bend voltage: 1500 V, fragmentary: 280 V, collision energy: 60 eV. The ion trap was operated over the scan range of *m*/*z* 100–1.700 for MS and MS/MS.

The chemical constituents were identified by comparing their retention index, mass spectra, and MS fragmentation with an in-house self-built database (Far-Eastern Agriculture Research Institute, Vostochnoe, Russia). The in-house self-built database was based on data from other spectroscopic techniques, such as nuclear magnetic resonance, ultraviolet spectroscopy, and MS, as well as data from the literature, which is continuously updated and revised. Data acquisition was controlled by Windows software for BRUKER DALTONIKS 2.0. The capture rate was one spectrum per second for MS and two spectra per second for MS/MS. Data collection was controlled using Bruker Daltonics software for Windows. All experiments were performed in triplicate. A four-stage ion separation mode (MS/MS) was implemented.

### 4.9. Statistical Analysis

All measured characteristics of the soybean cultivars were subjected to statistical analysis. In each trial, the order of samples was randomized, and analyses were performed in triplicate for each cultivar. Analysis of variance (ANOVA) was conducted using a completely randomized design. Differences among cultivars, concentrations, and their interactions were determined using Tukey’s range test at a significance level of *p* ≤ 0.05.

## 5. Conclusions

It has been established that Khabarovsk soybean varieties exhibit high variability in I, as well as in total phenolic and flavonoid content. The vegetable variety Kobra demonstrates the highest values for both AGP and seeds. Edible soybean varieties also differ in the I of their AGP and seeds, with values varying by approximately 3-fold between the minimum and maximum levels. Among the edible varieties, Khabarovskij yubilar exhibits the highest I. Our findings demonstrate the potential for breeding varieties suitable for cultivation in the southern regions of the Russian Far East and the northeastern regions of China. This work may also contribute to the development of functional food products and animal feed by utilizing by-products of varieties with higher levels of phenols, flavonoids, and antioxidant activity.

## Figures and Tables

**Table 1:** Proximate analyses of seeds of the eight soybean genotypes.

Cultivars	Nitrogen (%)	Crude Protein (%)	Fat (%)	Moisture (%)
Batya	6.25 ± 0.27	39.5 ± 0.6	19.4 ± 1.2	6.32 ± 0.75
Marinata	6.15 ± 0.34	38.2 ± 1.4	20.9 ± 1.4	6.35 ± 0.37
Soyuznaya 17	6.22 ± 0.19	38.8 ± 1.8	20.0 ± 1.2	6.48 ± 0.25
Zhemchuzhina vostoka	6.23 ± 0.10	38.1 ± 3.1	19.1 ± 2.1	6.41 ± 0.50
Kobra	6.11 ± 0.46	38.1 ± 1.6	20.0 ± 0.9	6.65 ± 0.12
Tasia	5.86 ± 0.62	37.4 ± 2.9	20.8 ± 1.2	6.37 ± 0.50
Khekhcirskaya	6.20 ± 0.48	39.6 ± 1.2	20.4 ± 1.1	6.55 ± 0.12
Khabarovskij yubilyar	6.03 ± 0.37	39.7 ± 0.5	20.0 ± 0.8	6.53 ± 0.25
$\bar{x}$	6.13	38.7	20.1	6.46
*σ*	0.13	0.9	0.6	0.11
$\Delta \bar{x}$	0.11	0.7	0.5	0.09
*CV*%	2.10	2.3	3.1	1.80
*p*-value	>0.05	>0.05	<0.01	>0.05

All data are expressed as the mean (*n* = 3) with ± Δ$\bar{x}$ in the same column; *CV*: coefficient of variation.

**Table 2:** Total phenol content, total flavonoid content and antioxidant activity of AGP of the eight soybean genotypes.

Cultivars	TPC (%)	TFC (%)	I (%)
Batya	10.5 ± 0.94	1.06 ± 0.02	44.9 ± 1.8
Marinata	5.82 ± 0.81	0.34 ± 0.04	16.1 ± 1.9
Soyuznaya 17	8.06 ± 0.40	0.78 ± 0.02	23.5 ± 0.7
Zhemchuzhina vostoka	10.8 ± 0.10	1.19 ± 0.04	31.3 ± 7.2
Kobra	10.2 ± 0.10	1.24 ± 0.08	54.3 ± 2.7
Tasia	8.59 ± 0.33	0.74 ± 0.09	31.3 ± 3.3
Khekhcirskaya	9.12 ± 0.21	1.13 ± 0.06	25.4 ± 1.4
Khabarovskij yubilyar	10.3 ± 0.83	1.18 ± 0.03	56.5 ± 1.9
$\bar{x}$	9.17	0.96	35.4
*σ*	1.65	0.31	14.8
$\Delta \bar{x}$	1.5	0.26	12.4
*CV*%	18.0	32.9	41.9
*p*-value	<0.0001	<0.0001	<0.0001

All data are expressed as the mean (*n* = 3) with ± Δ$\bar{x}$ in the same column; TPC: total phenolic content; TFC: total flavonoid content; I: antioxidant activity; *CV*: coefficient of variation.

**Table 3:** Total phenol content, total flavonoid content and antioxidant activity of the seeds of the eight soybean genotypes.

Cultivars	TPC (%)	TFC (%)	I (%)
Batya	2.56 ± 0.10	0.030 ± 0.002	12.7 ± 1.2
Marinata	2.62 ± 0.05	0.020 ± 0.002	22.7 ± 0.3
Soyuznaya 17	2.24 ± 0.07	0.030 ± 0.001	14.7 ± 1.7
Zhemchuzhina vostoka	2.68 ± 0.17	0.037 ± 0.004	23.5 ± 2.5
Kobra	4.44 ± 0.02	0.070 ± 0.006	47.5 ± 2.0
Tasia	2.55 ± 0.01	0.032 ± 0.004	24.7 ± 1.0
Khekhcirskaya	2.64 ± 0.14	0.026 ± 0.001	21.3 ± 0.3
Khabarovskij yubilyar	2.98 ± 0.29	0.036 ± 0.002	36.9 ± 1.2
$\bar{x}$	2.84	0.036	25.5
*σ*	0.68	0.020	11.5
Δ$\bar{x}$	0.57	0.010	9.6
*CV*%	23.9	43.0	45.1
*p*-value	<0.0001	<0.0001	<0.001

All data are expressed as the mean (*n* = 3) with ± $\Delta \bar{x}$ in the same column; TPC: total phenolic content; TFC: total flavonoid content; I: antioxidant activity; *CV*: coefficient of variation.

**Table 4:** Compounds detected in soybean AGP extract. Fragments were obtained at positive ionization mode and a relative collision energy of 60%. Diagnostic fragments of characteristic ions are marked in bold.

	Retention Time Window, min	[M + H]^+^	Cultivars	Main Fragments Obtained in Cultivars	Compound Identification [9,36]
1	21.9–24.5	255	Batya	**255**, **237**, **199**, 137	Daidzein
			Marinata	**255**, **237**, 171, 157, 145	
			Soyuznaya 17	**255**, **237**, **199**, 181, 145, 137	
			Zhemchuzhina vostoka	**255**, 236, 198, 157, 137	
			Kobra	**255**, **237**, **199**, 153, 137	
			Tasia	**255**, **237**, **199**, 156, 145, 137	
			Khekhcirskaya	**255**, **237**, **199**, 180, 156, 137	
			Khabarovskij yubilyar	**255**, **237**, **199**, 181, 153, 145, 137	
2	21.3–22.7	285	Batya	**285**, 229, 214, 186	Glycitein
			Soyuznaya 17	**285**, 229, 214	
			Tasia	**285**, **270**, **242**, 225, 214, 197, 169, 145	
			Khabarovskij yubilyar	**285**, **270**, 253, **242**, 169	
3	23.2–25.7	271	Batya	**271**, **253**, **215**, 187, 189, 153, 145	Genistein
			Marinata	**271**, **253**, 131	
			Soyuznaya 17	**271**, 243, **215**, 197, 153, 149	
			Zhemchuzhina vostoka	**271**, **253**, 243, **215**, 153, 197	
			Kobra	**271**, **253**, **215**, 224, 196, 153	
			Tasia	**271**, **253**, **215**, 153	
			Khekhcirskaya	**271**, **253**, 243, 225, 214, 186, 153, 149	
			Khabarovskij yubilyar	**271**, **253**, 243, 214, 197, 225, 187, 159, 131	

**Table 5:** Compounds detected in soybean seed extract. Fragments were obtained at positive ionization mode and a relative collision energy of 60%. Diagnostic fragments of characteristic ions are marked in bold.

	Retention Time Window, min	[M + H]^+^	Cultivars	Main Fragments Obtained in Cultivars	Compound Identification [9,36]
1	21.2–23.8	255	Batya	**255**, **199**, 119, 137	Daidzein
			Marinata	**255**, **237**, **199**, 181, 153, 137	
			Soyuznaya 17	**255**, **237**, **199**	
			Zhemchuzhina vostoka	**255**, **237**, **199**, 181	
			Kobra	**255**, **237**, 196, 181	
			Tasia	**255**, **237**, **199**, 171	
			Khekhcirskaya	**255**, **237**, 136, **199**, 156, 144, 118	
			Khabarovskij yubilyar	255, 237, 198, 181	
2	22.6–24.2	285	Batya	**285**, **270**, 225	Glycitein
			Marinata	**285**, **270**, **242**, 229	
			Soyuznaya 17	**285**, **270**, 253, 225, 145	
			Zhemchuzhina vostoka	**285**, **270**, 253, **242**	
			Kobra	**285**, **270**, 253, **242**, 225, 197, 145	
			Tasia	**285**, **270**, **242**, 225, 196, 144	
			Khekhcirskaya	**285**, **270**, 253, **242**, 229, 145	
			Khabarovskij yubilyar	**285**, **270**, 253, **242**, 225, 197, 169, 145	
3	25.2–27.3	271	Batya	**271**, **253**, 214, 153, 149	Genistein
			Marinata	**271**, **253**, 243, **215**, 186, 148	
			Soyuznaya 17	**271**, **253**, **215**, 144	
			Zhemchuzhina vostoka	**271**, **253**, 225, 197, 148	
			Kobra	**271**, **253**, 243, 224, 214, 196, 186, 159, 118	
			Tasia	**271**, **253**, **215**, 187, 149, 121	
			Khekhcirskaya	**271**, **253**, 243, 225, 214, 158, 118	

**Table 6:** Compounds detected in soybean AGP extract. Fragments were obtained at negative ionization mode and a relative collision energy of 60%. Diagnostic fragments of characteristic ions are marked in bold.

	Retention Time Window, min	[M − H]^−^	Cultivars	Main Fragments Obtained in Cultivars	Compound Identification [9,36]
1	19.5–21.9	353	Batya	**353**, **190**	Chlorogenic acid
			Soyuznaya 17	**353**, **190**	
			Zhemchuzhina vostoka	**353**, **190**	
			Tasia	**353**, **190**	
			Khekhcirskaya	**253**, **190**	
			Khabarovskij yubilyar	**353**, **190**	
2	6.6–8.8	289	Batya	**289**, **241**	(+)-Catechin
			Marinata	**289**, **245**	
			Soyuznaya 17	**289**, **245**	
			Zhemchuzhina vostoka	**289**, **245**	
			Kobra	**289**, **245**, 177	
			Tasia	**289**, **245**	
			Khekhcirskaya	**289**	
			Khabarovskij yubilyar	**289**, **245**	
3	20.9–27.3	179	Batya	**179**	Caffeic acid
			Soyuznaya 17	**179**	
			Zhemchuzhina vostoka	**179**	
			Kobra	**179**	
			Khekhcirskaya	**179**, 136	

**Table 7:** Compounds detected in soybean seed extract. Fragments were obtained at negative ionization mode and a relative collision energy of 60%. Diagnostic fragments of characteristic ions are marked in bold.

	Retention Time Window, min	[M − H]^−^	Cultivars	Main Fragments Obtained in Cultivars	Compound Identification [9,36]
1	8.0–9.7	289	Batya	**289**, **245**, 179	(+)-Catechin
			Marinata	**289**, **245**	
			Soyuznaya 17	**289**, **245**	
			Zhemchuzhina vostoka	**289**, **245**, 178	
			Kobra	**289**, **245**, 178	
			Tasia	**289**, 178	
			Khekhcirskaya	**289**, **245**	
			Khabarovskij yubilyar	**289**, **245**	
2	20.9–26.9	353	Batya	**353**, 190	Chlorogenic acid
			Marinata	**353**, 190	
			Zhemchuzhina vostoka	**353**, 190	
			Tasia	**353**, 190	
			Khekhcirskaya	**353**, 190	
			Khabarovskij yubilyar	**353**, 190	
3	24.3–26.4	179	Marinata	**179**, 134	Caffeic acid
			Soyuznaya 17	**179**, 134	
			Zhemchuzhina vostoka	**179**, 134	
			Kobra	**179**, 134	
			Khekhcirskaya	**179**, 133	
			Khabarovskij yubilyar	**179**, 134	

**Table 8:** Peak areas of total ion chromatograms of seeds and AGP of eight soybean varieties.

Cultivars	Seeds		AGP	
	S_TIC+_, mAU2 × 10^7^	S_TIC−_, mAU2 × 10^6^	S_TIC+_, mAU2 × 10^7^	S_TIC−_, mAU2 × 10^6^
Batya	64.7	33.3	117.2	64.4
Marinata	97.8	32.7	46.1	29.6
Soyuznaya 17	62.3	31.1	74.0	52.1
Zhemchuzhina vostoka	37.9	32.3	59.2	36.4
Kobra	148.3	55.8	111.6	51.8
Tasia	38.5	27.5	88.2	49.3
Khekhcirskaya	63.3	37.5	76.6	54.1
Khabarovskij yubilyar	117.5	51.7	94.3	70.5
$\bar{x}$	78.8	37.7	83.4	51.0
*σ*	39.1	10.3	24.5	13.3
*Δ* $\bar{x}$	32.7	8.6	20.5	11.2
*CV*%	49.6	27.3	29.3	26.1

*CV*: coefficient of variation.

**Table 9:** Correlation analysis among studied characteristics of soybeans AGP.

	TPC	TFC	I	S_TIC+_	S_TIC−_
TPC	-	0.94 ^a^	0.75 ^b^	0.63 ^c^	0.57 ^c^
TFC	0.94 ^a^	-	0.71 ^c^	0.58 ^c^	0.60 ^c^
I	0.75 ^b^	0.71 ^c^	-	0.81 ^b^	0.71 ^c^
S_TIC+_	0.63 ^c^	0.58 ^c^	0.81 ^b^	-	0.89 ^a^
S_TIC−_	0.57 ^c^	0.60 ^c^	0.71 ^c^	0.89 ^a^	-

a, b, c represent Pearson’s correlation coefficients (r) at *p* < 0.01, <0.05 and *p* > 0.05, respectively. The scatterplot visualized all pairs of correlations shown in Figure S1, Supplementary files.

**Table 10:** Correlation analysis among studied characteristics of soybean seeds.

	TPC	TFC	I	S_TIC+_	S_TIC−_
TPC	-	0.92 ^a^	0.90 ^a^	0.80 ^b^	0.84 ^b^
TFC	0.92 ^a^	-	0.83 ^b^	0.61 ^c^	0.73 ^b^
I	0.90 ^a^	0.83 ^b^	-	0.78 ^b^	0.87 ^b^
S_TIC+_	0.80 ^b^	0.61 ^c^	0.78 ^b^	-	0.79 ^b^
S_TIC−_	0.84 ^a^	0.73 ^b^	0.87 ^b^	0.79 ^b^	-

a, b, c represent Pearson’s correlation coefficients (r) at *p* < 0.01, <0.05 and *p* > 0.05, respectively. The scatterplot visualized all pairs of correlations shown in Figure S2, Supplementary files.

**Table 11:** Origin and pedigree for the eight soybean cultivars.

№	Seed Color	Accession	Origin	Pedigree
1	Yellow	Batya	Russia	Midori Supporo × Vaz-100
2	Yellow	Marinata	Russia	Yug-40 × Saltus
3	Yellow	Soyuznaya 17	Russia	Selected from Dunchuan 2
4	Yellow	Zhemchuzhina vostoka	Russia	Selected from *Glycine hispida* (Moench) Max (L.) ssp. Koraiensis Enk.
5	Black	Kobra	Russia	Selected from Amurskaja 41
6	Yellow	Tasia	Russia	Selected from Dunchuan 1
7	Yellow	Khekhcirskaya	Russia	Selected from Dunchuan 3
8	Yellow	Khabarovskij yubilyar	Russia	Sonata × Marinata

## Data Availability

The original contributions presented in this study are included in the article and Supplementary Information.

## Supplementary Materials

The following supporting information can be downloaded at: https://www.mdpi.com/article/10.3390/molecules31152671/s1, Table S1: Confidence probabilities for Tukey’s method (Seeds; Fat,%); Table S2: Confidence probabilities for Tukey’s method (AGP; TPC,%); Table S3: Confidence probabilities for Tukey’s method (AGP; TFC,%); Table S4: Confidence probabilities for Tukey’s method (AGP; I,%); Table S5: Confidence probabilities for Tukey’s method (Seeds; TPC,%); Table S6: Confidence probabilities for Tukey’s method (Seeds; TFC,%); Table S7: Confidence probabilities for Tukey’s method (Seeds; I,%); Figure S1: The scatterplot showing all pairs of correlations in studied characteristics of soybean AGP; Figure S2: The scatterplot showing all pairs of correlations in studied characteristics of soybean seeds; Figure S3: Eight soybean cultivars seeds: (a) Batya; (b) Marinata; (c) Soyuznaya 17; (d) Zhemchuzhina vostoka; (e) Kobra; (f) Tasia; (g) Khekhcirskaya; (h) Khabarovskij yubilyar; Figure S4: Eight soybean cultivars above-ground parts: (a) Batya; (b) Marinata; (c) Soyuznaya 17; (d) Zhemchuzhina vostoka; (e) Kobra; (f) Tasia; (g) Khekhcirskaya; (h) Khabarovskij yubilyar.
