# Supplementary material for: Determination of Phenolic Content and Antioxidant Activity in Khabarovsk Soybean Cultivars
Source: Molecules. 2026 Jul 31;31(15):2671. doi: 10.3390/molecules31152671 (PMC13467611; doi:10.3390/molecules31152671)
Supplement: Supplementary file 1 [file molecules-31-02671-s001.zip › molecules-4415228-supplementary.pdf]

Table S1. Confidence probabilities for Tukey's method (Seeds; Fat,%).

Cultivars	Batya	Marinata	Soyuznaya 17	Zhemchuzhina vostoka	Kobra	Tasia	Khekhcirskaya	Khabarovskij yubilyar
Batya	-	**P<0.05**	P>0.05	P>0.05	P>0.05	P>0.05	P>0.05	P>0.05
Marinata	**P<0.05**	-	P>0.05	**P<0.01**	P>0.05	P>0.05	P>0.05	P>0.05
Soyuznaya 17	P>0.05	P>0.05	-	P>0.05	P>0.05	P>0.05	P>0.05	P>0.05
Zhemchuzhina vostoka	P>0.05	**P<0.01**	P>0.05	-	P>0.05	**P<0.05**	P>0.05	P>0.05
Kobra	P>0.05	P>0.05	P>0.05	P>0.05	-	P>0.05	P>0.05	P>0.05
Tasia	P>0.05	P>0.05	P>0.05	**P<0.05**		-	P>0.05	P>0.05
Khekhcirskaya	P>0.05	P>0.05	P>0.05	P>0.05	P>0.05		-	P>0.05
Khabarovskij yubilyar	P>0.05	P>0.05	P>0.05	P>0.05	P>0.05	P>0.05	P>0.05	-

Table S2. Confidence probabilities for Tukey's method (AGP; TPC,%).

Cultivars	Batya	Marinata	Soyuznaya 17	Zhemchuzhina vostoka	Kobra	Tasia	Khekhcirskaya	Khabarovskij yubilyar
Batya	-	**P<0.001**	**P<0.001**	P>0.05	P>0.05	**P<0.001**	**P<0.001**	P>0.05
Marinata	**P<0.001**	-	**P<0.001**	**P<0.001**	**P<0.001**	**P<0.001**	**P<0.001**	**P<0.001**
Soyuznaya 17	**P<0.001**	**P<0.001**	-	**P<0.001**	**P<0.001**	P>0.05	**P<0.05**	**P<0.001**
Zhemchuzhina vostoka	P>0.05	**P<0.001**	**P<0.001**	-	**P<0.05**	**P<0.001**	**P<0.001**	**P<0.05**
Kobra	P>0.05	**P<0.001**	**P<0.001**	**P<0.05**	-	**P<0.001**	**P<0.01**	P>0.05
Tasia	**P<0.001**	**P<0.001**	P>0.05	**P<0.001**	**P<0.001**	-	P>0.05	**P<0.001**
Khekhcirskaya	**P<0.001**	**P<0.001**	**P<0.05**	**P<0.001**	**P<0.01**	P>0.05	-	**P<0.01**
Khabarovskij yubilyar	P>0.05	**P<0.001**	**P<0.001**	**P<0.05**	P>0.05	**P<0.001**	**P<0.01**	-

Table S3. Confidence probabilities for Tukey's method (AGP; TFC,%).

Cultivars	Batya	Marinata	Soyuznaya 17	Zhemchuzhina vostoka	Kobra	Tasia	Khekhcirskaya	Khabarovskij yubilyar
Batya	-	**P<0.001**	**P<0.001**	**P<0.001**	**P<0.001**	**P<0.001**	**P<0.05**	**P<0.01**
Marinata	**P<0.001**	-	**P<0.001**	**P<0.001**	**P<0.001**	**P<0.001**	**P<0.001**	**P<0.001**
Soyuznaya 17	**P<0.001**	**P<0.001**	-	**P<0.001**	**P<0.001**	P>0.05	**P<0.001**	**P<0.001**
Zhemchuzhina vostoka	**P<0.01**	**P<0.001**	**P<0.001**	-	P>0.05	**P<0.001**	P>0.05	P>0.05
Kobra	**P<0.001**	**P<0.001**	**P<0.001**	P>0.05	-	**P<0.001**	**P<0.01**	**P<0.05**
Tasia	**P<0.001**	**P<0.001**	P>0.05	**P<0.001**	**P<0.001**	-	**P<0.001**	**P<0.001**
Khekhcirskaya	**P<0.05**	**P<0.001**	**P<0.001**	P>0.05	**P<0.01**	**P<0.001**	-	P>0.05
Khabarovskij yubilyar	**P<0.01**	**P<0.001**	**P<0.001**	P>0.05	**P<0.05**	**P<0.001**	P>0.05	-

Table S4. Confidence probabilities for Tukey's method (AGP; I,%).

Cultivars	Batya	Marinata	Soyuznaya 17	Zhemchuzhina vostoka	Kobra	Tasia	Khekhcirskaya	Khabarovskij yubilyar
Batya	-	**P<0.001**	**P<0.001**	**P<0.001**	**P<0.001**	**P<0.001**	**P<0.001**	**P<0.001**
Marinata	**P<0.001**	-	**P<0.001**	**P<0.001**	**P<0.001**	**P<0.001**	**P<0.001**	**P<0.001**
Soyuznaya 17	**P<0.001**	**P<0.001**	-	**P<0.001**	**P<0.001**	**P<0.001**	P>0.05	**P<0.001**
Zhemchuzhina vostoka	**P<0.001**	**P<0.001**	**P<0.001**	-	**P<0.001**	P>0.05	**P<0.01**	**P<0.001**
Kobra	**P<0.001**	**P<0.001**	**P<0.001**	**P<0.001**	-	**P<0.001**	**P<0.001**	P>0.05
Tasia	**P<0.001**	**P<0.001**	**P<0.001**	P>0.05	**P<0.001**	-	**P<0.01**	**P<0.001**
Khekhcirskaya	**P<0.001**	**P<0.001**	P>0.05	**P<0.01**	**P<0.001**	**P<0.01**	-	**P<0.001**

Khabarovskij yubilyar	**P<0.001**	**P<0.001**	**P<0.001**	**P<0.001**	P>0.05	**P<0.001**	**P<0.001**	-

Table S5. Confidence probabilities for Tukey's method (Seeds; TPC,%).

Cultivars	Batya	Marinata	Soyuznaya 17	Zhemchuzhina vostoka	Kobra	Tasia	Khekhcirskaya	Khabarovskij yubilyar
Batya	-	P>0.05	**P<0.001**	P>0.05	**P<0.001**	P>0.05	P>0.05	**P<0.001**
Marinata	P>0.05	-	**P<0.001**	P>0.05	**P<0.001**	P>0.05	P>0.05	**P<0.001**
Soyuznaya 17	**P<0.001**	**P<0.001**	-	**P<0.001**	**P<0.001**	**P<0.001**	**P<0.001**	**P<0.001**
Zhemchuzhina vostoka	P>0.05	P>0.05	**P<0.001**	-	**P<0.001**	P>0.05	P>0.05	**P<0.001**
Kobra	**P<0.001**	**P<0.001**	**P<0.001**	**P<0.001**	-	**P<0.001**	**P<0.001**	**P<0.001**
Tasia	P>0.05	P>0.05	**P<0.001**	P>0.05	**P<0.001**	-	P>0.05	**P<0.001**
Khekhcirskaya	P>0.05	P>0.05	**P<0.001**	P>0.05	**P<0.001**	P>0.05	-	**P<0.001**
Khabarovskij yubilyar	**P<0.001**	**P<0.001**	**P<0.001**	**P<0.001**	**P<0.001**	**P<0.001**	**P<0.001**	-

Table S6. Confidence probabilities for Tukey's method (Seeds; TFC,%).

Cultivars	Batya	Marinata	Soyuznaya 17	Zhemchuzhina vostoka	Kobra	Tasia	Khekhcirskaya	Khabarovskij yubilyar
Batya	-	**P<0.001**	P>0.05	**P<0.01**	**P<0.001**	P>0.05	P>0.05	**P<0.01**
Marinata	**P<0.01**	-	**P<0.01**	**P<0.001**	**P<0.001**	**P<0.001**	P>0.05	**P<0.001**
Soyuznaya 17	P>0.05	**P<0.01**	-	**P<0.01**	**P<0.001**	P>0.05	P>0.05	**P<0.05**
Zhemchuzhina vostoka				-	**P<0.001**	P>0.05	**P<0.001**	P>0.05
Kobra	**P<0.001**	**P<0.001**	**P<0.001**	**P<0.001**	-	**P<0.001**	**P<0.001**	**P<0.001**
Tasia	P>0.05	**P<0.001**	P>0.05	P>0.05	**P<0.001**	-	**P<0.01**	P>0.05
Khekhcirskaya	P>0.05	P>0.05	P>0.05	**P<0.001**	**P<0.001**	**P<0.01**	-	**P<0.001**
Khabarovskij yubilyar	**P<0.01**	**P<0.001**	**P<0.05**	P>0.05	**P<0.001**	P>0.05	**P<0.001**	-

Table S7. Confidence probabilities for Tukey's method (Seeds; I,%).

Cultivars	Batya	Marinata	Soyuznaya 17	Zhemchuzhina vostoka	Kobra	Tasia	Khekhcirskaya	Khabarovskij yubilyar
Batya	-	**P<0.001**	**P<0.05**	**P<0.001**	**P<0.001**	**P<0.001**	**P<0.001**	**P<0.001**
Marinata	**P<0.001**	-	**P<0.001**	P>0.05	**P<0.001**	**P<0.05**	P>0.05	**P<0.001**
Soyuznaya 17	**P<0.05**	**P<0.001**	-	**P<0.001**	**P<0.001**	**P<0.001**	**P<0.001**	**P<0.001**
Zhemchuzhina vostoka	**P<0.001**	P>0.05	**P<0.001**	-	**P<0.001**	P>0.05	**P<0.01**	**P<0.001**
Kobra	**P<0.001**	**P<0.001**	**P<0.001**	**P<0.001**	-	**P<0.001**	**P<0.001**	**P<0.001**
Tasia	**P<0.001**	**P<0.05**	**P<0.001**	P>0.05	**P<0.001**	-	**P<0.001**	**P<0.001**
Khekhcirskaya	**P<0.001**	P>0.05	**P<0.001**	**P<0.01**	**P<0.001**	**P<0.001**	-	**P<0.001**
Khabarovskij yubilyar	**P<0.001**	**P<0.001**	**P<0.001**	**P<0.001**	**P<0.001**	**P<0.001**	**P<0.001**	-

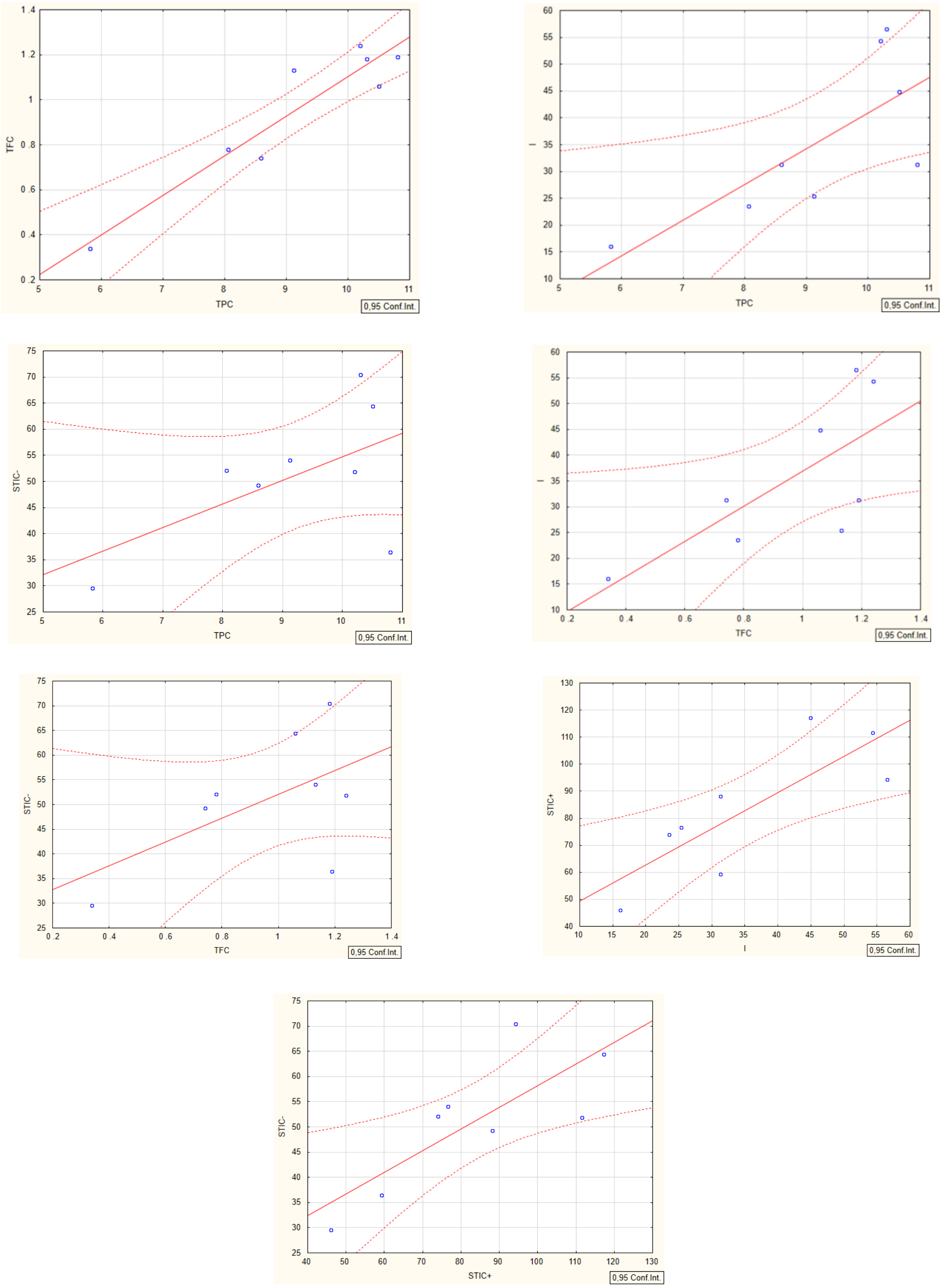

Figure S1. The scatterplot showing all pairs of correlations in studied characteristics of soybean AGP.

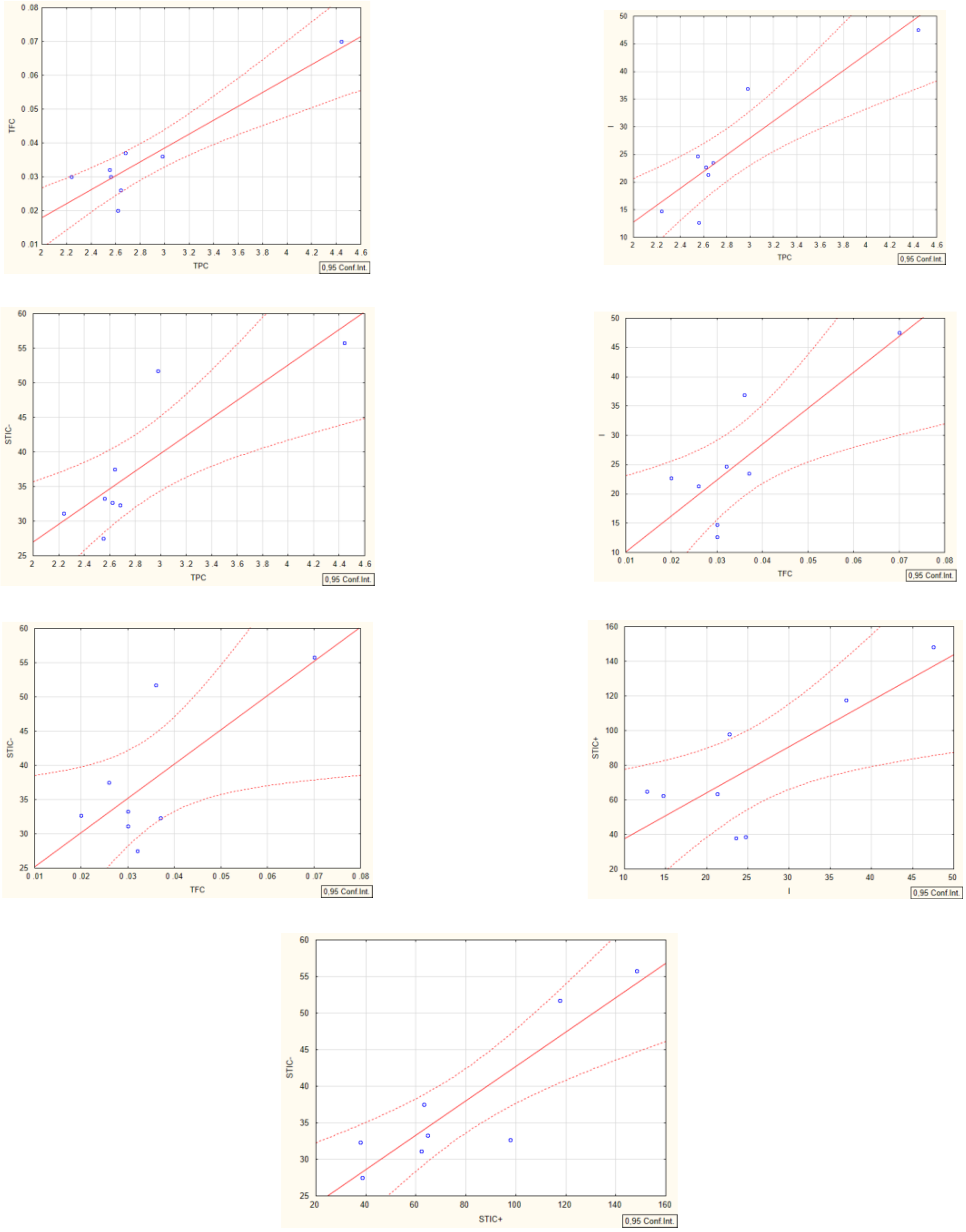

Figure S2. The scatterplot showing all pairs of correlations in studied characteristics of soybean seeds.

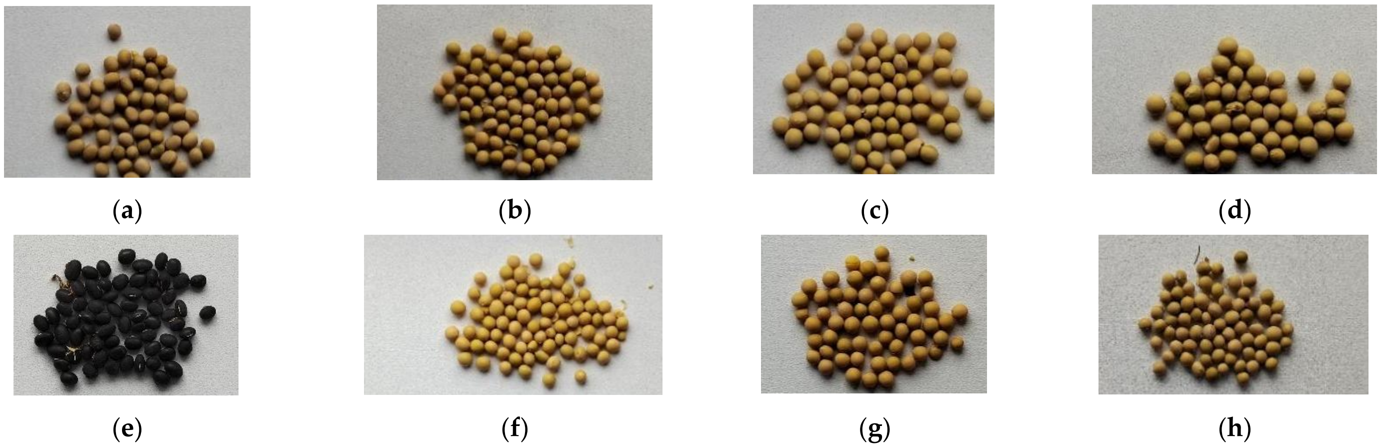

Figure S3. Eight soybean cultivars seeds: (a) Batya; (b) Marinata; (c) Soyuznaya 17; (d) Zhemchuzhina vostoka; (e) Kobra; (f) Tasia; (g) Khekhcirskaya; (h) Khabarovskij yubilyar

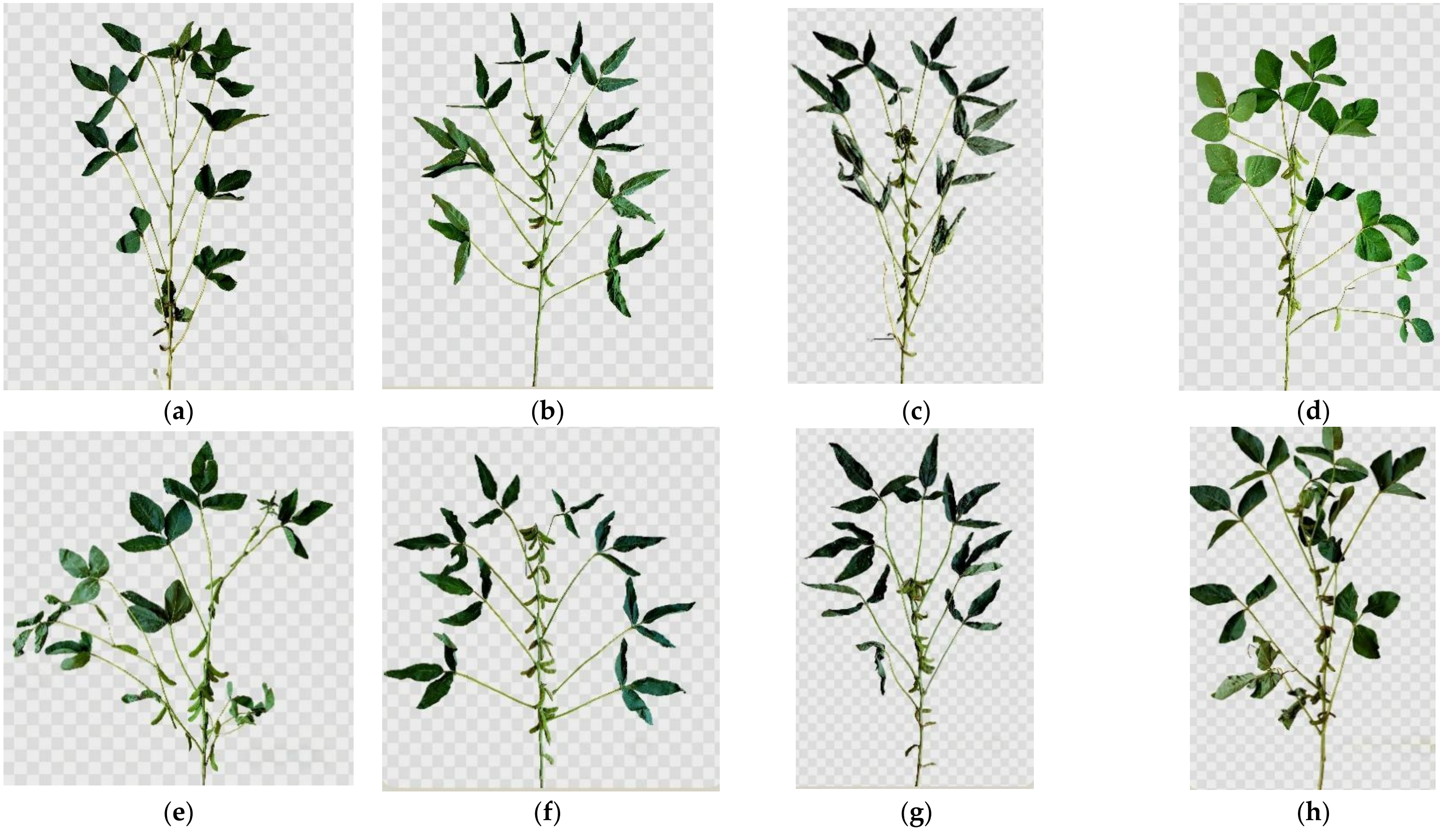

Figure S4. Eight soybean cultivars above-ground parts: (a) Batya; (b) Marinata; (c) Soyuznaya 17; (d) Zhemchuzhina vostoka; (e) Kobra; (f) Tasia; (g) Khekhcirskaya; (h) Khabarovskij yubilyar
